# Bacterial Profile, Antimicrobial Susceptibility Pattern, and Associated Factors of Community- and Hospital-Acquired Urinary Tract Infection at Dessie Referral Hospital, Dessie, Northeast Ethiopia

**DOI:** 10.1155/2021/5553356

**Published:** 2021-09-18

**Authors:** Berhanu Adugna, Bekele Sharew, Mohabaw Jemal

**Affiliations:** ^1^Dessie Referral Hospital, Laboratory Technologist, The Department of Medical Microbiology, Dessie, Ethiopia; ^2^Wollo University, Department of Medical Laboratory Sciences, Dessie, Ethiopia; ^3^University of Gondar, College of Medicine and Health Sciences, School of Biomedical and Laboratory Sciences, Department of Medical Microbiology, Gondar, Ethiopia

## Abstract

**Background:**

Bacterial urinary tract infection is among the most common community and hospital-acquired infections. Therefore, to know the status of the community and hospital-acquired urinary tract infection, antimicrobial susceptibility patterns, and associated factors among urinary tract infection profiles are essential to physicians and health workers to implement appropriate intervention.

**Methods:**

An institution-based cross-sectional study was conducted among 422 urinary tract infection suspected patients. All isolates were identified by standard microbiological techniques, and their antibiotic susceptibility was done by the Kirby–Bauer disc diffusion method. Data were entered using EpiData version 3.1 and analyzed by SPSS software version 20. *P* value < 0.05 at 95% CI was considered statistically significant.

**Result:**

Of 422 urine samples processed, 100 (23.7%) yielded bacterial isolates. About 50(30.7%) and 50(19.3%) were bacterial isolates from the community and hospitalized patients, respectively. *E. coli* 44/103(42.7%) predominated across the two groups, followed by *S. aureus* 25/103(24.3%), CONs, 14/103(13.5%), *Klebsiella* spp. 7/103(6.78), *Proteus* spp. 3/103(2.91), and *Enterococcus* spp. 3/103 (2.91%). *Pseudomonas* spp. 3/103 (2.91), *Citrobacter* spp. 2/103(1.94%), and *Acinetobacter* spp. 1/103(0.999), which were isolated from only the hospitalized patients. Meropenem susceptibly was 100% in both study groups and Ampicillin resistance was documented as 83.3% to 100% and 76.9% to 100% in hospitalized and community-acquired samples, respectively.

**Conclusion:**

This study found a high prevalence of bacterial urinary tract infection in the study area and a high rate of bacterial resistance was observed to most antimicrobial drugs tested. Meropenem and nitrofurantoin were the most active drugs for urinary tract infections. Therefore, expanding routine bacterial culture and identification with antimicrobial susceptibility testing and strengthening regular surveillance systems are essential for appropriate patient care.

## 1. Introduction

Urinary tract infections (UTI) constitute a significant public health problem and present an important cause of morbidity and mortality worldwide [1, 2]. Bacterial pathogens are the commonest etiological agent to cause UTI. It affects both lower and upper urinary tracts with different clinical symptoms, including fever, dysuria, urgency, burning sensation, and intermittent urination.

Suprapubic tenderness [3] is the second most common infection after respiratory tract infections and accounts for a great proportion of prescriptions of antibiotics. The major causative organisms for UTI are bacteria organisms. They account for more than 95% of cases [4, 5], which may be Gram-negative and Gram-positive bacteria that account for 80–85% and 15–20%, respectively [6, 7]. Urinary tract infection starts with contamination of the periurethral by uropathies residing in the bowel flora colonization. The urethra ascends to the bladder and migrates to the kidney or prostate. The result of host-pathogen complex interactions ultimately determines whether uropathogens are successful in colonization or eliminated [8]. Community-acquired UTI (CAUTI) is the member of intestinal microbial flora. The most common are *E. coli* and *Klebsiella* spp. In community-acquired urinary tract infection, *E. coli* and *S. saprophyticus* accounts for 80% and 5% to 15% of outpatients, respectively, across the various regions of the world, and the remaining 5% to 10% of cases are aerobic Gram-negative rods such as *Klebsiella* spp. *and Proteus* spp. and other *Enterobacter* spp. [9–11].

Hospital-acquired urinary tract (HAUTI) infections are mostly healthcare-related infections because these are the ones that occur more frequently and are commonly related to the use of a medical device such as catheterization [10, 12, 13].

Urinary tract infections are an important cause of septicemia, resulting in high mortality rates, prolonged hospital stays, and increased healthcare costs [14]. The death of hospitalized patients among the victims of hospital-acquired urinary tract infections are 2 to 3-times higher than those among nonbacteriuric patients [15]. Catheter-associated urinary tract infections occur with high incidence if healthcare safety is not maintained. Studies reveal that 79.3% of UTI can be prevented if catheterization is not performed in hospitals [16]. Multiple risk factors can affect the occurrence of urinary tract infections. These include age, sex, catheterization and hospitalization, previous exposure to antibiotics, recurrent UTI, duration of catheterization, and care of catheter [12, 17–20].

In developing countries, including Ethiopia, where there is a high level of poverty and poor hygiene practices, there is also a high prevalence of fake and spurious drugs of questionable quality in circulation [21, 22]. Besides this, the easy availability in the community without clinician order and low cost makes the drugs subject to abuse. These make increasing drug resistance [23]. Therefore, the current study aims to assess the prevalence and antimicrobial susceptibility pattern and associated factors among urinary tract infection profiles and provides updated information to regulatory bodies and those who would like to use the findings for the development of intervention strategies as appropriate.

This manuscript is presented in the < research square > as a preprint. https://www.researchsquare.com/article/rs-157817/v1.

## 2. Materials and Methods

### 2.1. Study Design and Setting

A hospital-based cross-sectional study was conducted at Dessie Referral Hospital from March 2019- April 2019. The hospital was found in Dessie town with a distance of 401 km from the capital city of Ethiopia, Addis Ababa, and 471 km far from Bahir Dar, which is the capital city of Amhara Regional State. The hospital provides health services for more than 6 million people. This large number of people from the surrounding zones and nearby regions visits the hospital for different medical services. Dessie Referral Hospital provides emergency, ART services, chronic care, surgical, dental, medical, pediatric, gynecologic, obstetric, and other services.

### 2.2. Sample Size and Sampling Technique

A single population proportion formula was used to determine the sample size, 50% prevalence (anticipated proportion). By considering a 5% margin of error, 95% confidence level, and 10% nonresponse rate, a total of 422 participants were proposed and systematically recruited.

### 2.3. Data Collection Tools and Procedure

#### 2.3.1. Sociodemographic and Clinical Data

Data related to sociodemographic factors, clinical data, and associated factors were collected using pretested structured and standardized questionnaires from reviewed literature. They were prepared in English, translated to the local language (Amharic), and then translated back into English to check its consistency. Data were collected from the places of study participants from outpatient departments or inpatient wards.

### 2.4. Sample Collection and Transportation

A freshly voided midstream urine sample (10–15 ml) and catheterized urine samples were collected using a sterile container with screw cap tops. Urine samples were examined chemically and microscopically. Then, they were delivered to Amhara public health microbiology laboratory and processed within 1-2 hours for analysis. In case of delay, the samples were refrigerated at 2–8°C for up to 6 h [24].

### 2.5. Isolation and Identification Procedure

Urine specimens collected from different departments were directly inoculated by using calibrated inoculating wire loop (0.001 mL) on cystine lactose electrolyte deficient agar (CLED) (Oxoid Ltd., England). Culture plates were incubated in the aerobic environment at 370C for 24 hrs. After incubation, all suspected colonies were subcultured onto MacConkey agar (Oxoid, England) and 5% sheep blood agar (Oxoid, England) for further identification. All positive cultures were further identified by their colony characteristics, and Gram staining was done to identify Gram-positives from Gram-negatives. The biochemical tests used for final identification were Triple sugar iron agar test, Sulphide Indole production test, citrate utilization test, urease production test, and catalase and coagulase test [24].

### 2.6. Antimicrobial Susceptibility Testing

Antimicrobial susceptibility tests were carried out using the Kirby–Bauer disc diffusion method as per the Clinical Laboratory Standards Institute (CLSI) guidelines on Muller-Hinton agar. Pure colonies were taken from plates with fresh, pure culture using sterile wire loops and transferred to a tube containing 5 ml of 0.85% normal saline and mixed gently until it formed a homogeneous suspension. The turbidity of the suspension was then adjusted to the density of a McFarland standard 0.5 to standardize the size of the inoculum. The surface of the Muller-Hinton agar was then completely covered by rotating the swab with the suspension. The plates were allowed to dry for 3–5 minutes: then, discs were evenly distributed 24 mm apart on the inoculated plate using sterile forceps and incubated at 37 0C for 18–24 hours. The diameter of the zone of inhibition around the disc was measured using a ruler. Results were interpreted as Sensitive, Intermediate, and Resistant based on CLSI 2016 guideline [25]. The following routinely used antimicrobials were tested: ampicillin (AMP, 10 *µ*g), amoxclavunic (20/10), tetracycline (TE, 30 *µ*g), ciprofloxacin (CIP, 5 *µ*g), trimethoprim + sulphamethozol (SXT, 1.25/23.75), gentamicin (CN, 10 *µ*g), ceftriaxone (CRO, 30 *µ*g), cefixime (CXM, 5 *µ*g), nalidixic acid (NA, 30 *µ*g), nitrofurantoin (F, 300 *µ*g), piperacillin (PIP, 100 *µ*g), vancomycin (VAN, 10 *µ*g), penicillin (PEN, 10 *µ*g), meropenem (MER, 10 *µ*g), and tobramycin (TOB, 10 *µ*g), and then plates were incubated at 37°C for 24 h. Multidrug resistance was defined as the resistance of an isolate to three or more antimicrobial classes tested [25].

#### 2.6.1. Data Quality Control

The data collector was trained in the methods of data collection technique. The completeness and clarity of the collected data were checked every day. A pretested structured questionnaire was used for the data collection on sociodemographic characteristics and associated risk factors. The questionnaire was initially prepared in English and translated into the local language, Amharic.

#### 2.6.2. Laboratory Quality Control

The sterility of culture media was checked by incubating about 5% a batch of the media at 35–37OC overnight and evaluated for possible contamination. Standard reference strains of *S. typhimurium* (ATCC-14028) and *E. coli* (ATCC-25922) were used as quality control throughout the study for culture [26]. Data quality was ensured at various activities of the study by following the prepared standard operating procedure (SOP) of the laboratory.

### 2.7. Data Analysis and Interpretation

Collected data were entered into Epi-data 3.1 and exported to SPSS version 20 statistical software for analysis. During analysis, Descriptive statistics including mean, frequency, and percentage were used to summarize the data as appropriate. Then the findings of this study were presented in the form of texts, tables, and graphs as appropriate. A *P* value of <0.05 was considered statistically significant.

#### 2.7.1. Operational Definition

Community-acquired UTI is an infection if an individual with UTI is suspected before hospital admission and specimens are collected from the outpatient or within less than 48 hours of hospital admission.

#### 2.7.2. Hospital-Acquired UTI

Those individuals are not present or incubating at the time of the hospital admission and developing 48–72 hours after hospital admission. This manuscript presents in the <research square> as a preprint. https://www.researchsquare.com/article/rs-157817/v1.

## 3. Results

### 3.1. Sociodemographic and Clinical Characteristics

A total of 422 Urinary tract infection suspected patients were included in this study. Of these, 281 (66.6%) were female and 141 (33.4%) were male. The age range of study participants was 5–90 years, with a median age of 32 years. Among the total number of study subjects, 114 (27%), 110 (26%), and 66 (15.6%) were (30-44), (15-29), and (0–14) years of age, receptively. One hundred thirty-seven had an educational level of reading and writing only. The majority of the study subjects were from urban areas and had lower (<500EBR) monthly income, 238 (56.4%) and 214 (50.7%), respectively (Table 1). Moreover, 154 (36.4%), 72 (17%), 49 (11.6), and 40 (9.5%) had a history of previous exposure to antibiotics, diabetics (CDs), history of renal calculi, and history of urinary tract obstruction in the community and hospital-acquired UTI, respectively. Out of the total participants clinically diagnosed with urinary tract infection were studied to isolate bacteria from urine, of which 259 (61.4%) were from community-acquired cases and 163 (38.6%) from hospital-acquired cases [Table 1].

### 3.2. Prevalence of Urinary Tract Infections

**Figure 1:**
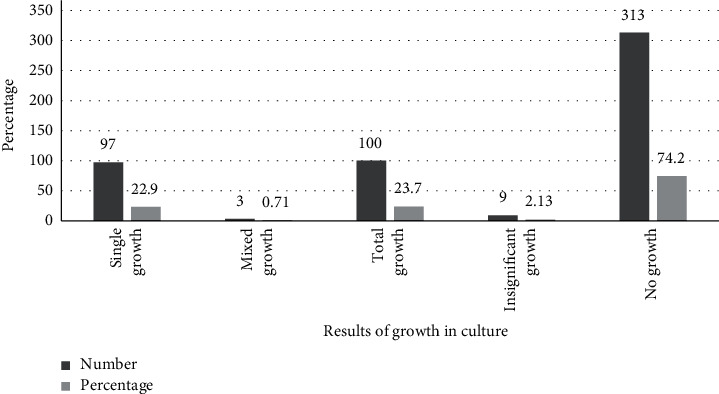
Bacterial growth results in the culture media from a total urine sample processed at Dessie Referral Hospital, Dessie.

**Figure 2:**
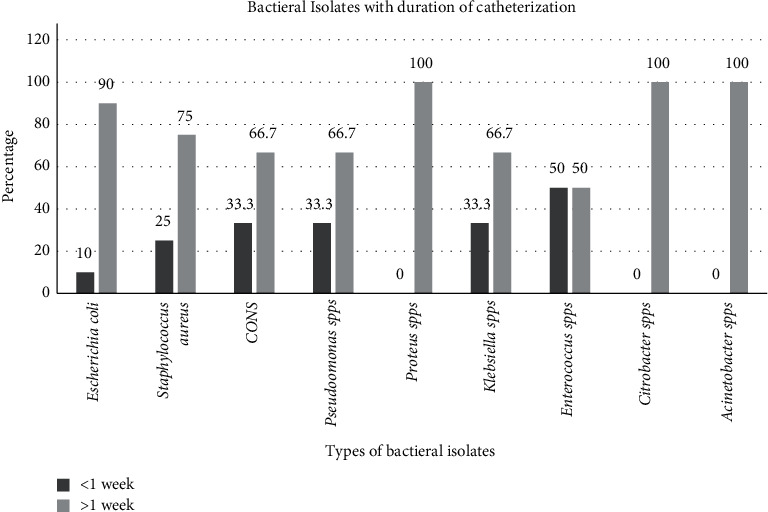
Distribution of isolates varies in the duration of catheterization at Dessie Referral Hospital.

The overall prevalence of urinary tract infection was 23.7% (100/422) (95% CI: 19.3–27.5). Out of 259 community-acquired UTI symptomatic patients, 19.3% (50/259) (95% CI:16.0–24.7) were culture-positive and 30.7% (50/163) (95% CI:23.3–38) were culture-positive for hospitalized patients (Table 2). Of the total 422 urine specimens processed, 74.2% (313/422) showed no bacteriuria growth and 2.13% (9/422) showed insignificant bacterial growth. Significant growth was present in 23.7% (100/422) samples with 22.99% (97/422) single growth and 0.71% (3/422) in mixed growth with two organisms in hospitalized patients (Figure 1). Three out of four hundred twenty-two (0.71%) samples with two bacteria each were isolated, making the number of bacteria isolated to be 103 with the isolation rate of (24.4%). From a total of 103 different uropathies bacterial isolated, 53 (51.46%) were hospital-acquired setting isolates, and 50(48.54% were community-acquired setting isolates (Table 3). Sixty-one (59.22%) were Gram-negative bacilli and 42(40.78%) were Gram-positive cocci (Figure 2).

The predominant bacteria isolated in both community and hospital-acquired UTI were *E. coli* 52%(26/50) versus 33.96% (18/53), followed by *S. aureus* 24% (12/50) vs. 24.5% (13/53), CONs16% (8/53) vs. 11.32% (6/53), *Klebsiella* spp. 4%(2/50) vs. 9.43% (5/53), *Proetus* spp. 2% (1/50) vs. 5.7%(3/53), and *Enterococcus* spp. 2%(1/50) vs. 3.8%(2/53) were isolated in both study groups whereas *Pseudomonas* spp. 5.7% (3/53), *Citrobacter* spp. 3.8% (2/53), and *Acinetobacter* spp. 1.79% (1/53) were isolated only in hospitalized patients (Table 3).

The frequency of isolated bacteria increases the duration of catheterization. *E. coli*, *S. aureus*, CONs, *Pseudomonas* spp., *Klebsiella*, and *Enterococcus* spp. were increased after one week of catheterization. However, *Proteus* spp.*, Citrobacter* spp.*, and Acinetobacter* spp. were found only for more than one week of catheterization (Figure 2).

### 3.3. Risk Factors of Community and Hospital-Acquired Urinary Tract Infection

In bivariate and multivariate analysis of CAUTI study subject, the previous usage of antibiotics was 4.427 times more likely to have developed urinary tract infection when compared with nonusers of antibiotics (AOR = 4.427; CI, 1.214–16.146, *P*=0.024). However, there was no association among other characteristics like sex, age, pregnancy, chronic disease, and recurrence of urinary tract infection (*P* value >0.05) (Table 4).

The prevalence of bacteria significantly differs from inpatient 53/163(30.7%) to outpatient 50/259 (19.3%) UTIs. (*X*2 = 6.537, OR = 1.753, CI:1.175–2.912, *P*=0.011) (Table 2). Those individuals who were inpatient were 1.753 times more likely exposed to develop HAUTIas compared to outpatient individuals. In addition, being female of sex was 8.925 times more likely to have increased urinary tract infection as compared with being male (AOR = 8.925; CI:1.790–44.48, *P*=0.008), and ages of 15–29 years and 30–45 years old were 0.126 and 0.057 less likely to have developed urinary tract infection when compared with ages of individuals whose age were >60 years old (AOR = 0.126; CI,0.020–0.792, *P*=0.027) and (AOR = 0.057; CI,0.057–0.480, *P*=0.008), respectively [Table 4].

In addition, individuals with the diabetic disease were 6.702 times more likely to have increased developing UTI as compared with individuals who were not diabetic (AOR = 6.702; CI,1.994–22.528, *P*=0.002) [Table 4].

Besides these, previous usage of prolonged antibiotics was 5.689 times more likely to have developed urinary tract infection when compared with nonusers of antibiotics (AOR = 5.689; CI.1.840–17.590, *P*=0.003). Moreover, those patients who used catheters were 3.886 times more likely to have increased developing UTI as compared with those patients who have not used catheters (AOR = 3.886; CI,1.323–11.47, *P*=0.014) (Table 4).

However, there was no association among other characteristics like ages 0–14 years and 45–59 years, recurrent urinary tract infection, history of urinary tract obstruction, history of renal calculi, waiting for time in hospital, and duration of catheterization (*P* value >0.05) (Table 4).

### 3.4. Antimicrobial Susceptibility of Community and Hospital-Acquired Urinary Tract Infection

In this study, the highest degree of resistance among the 15 antimicrobial drugs was observed indifferent bacterial isolates. *Klebsiella* spp. was resistant to 100% for Ampicillin and 80% Augmentin in hospital-acquired and Tetracycline in community-acquired UTI. Ampicillin, Tetracycline, and cotrimoxazole were resisted 100% by *Proetus* spp. in both study groups and Augmentin was 100% resisted in community-acquired UTI as well. *Citrobacter* spp. was 100% resistant to Augmentin, Tetracycline, Cotrimoxazole, and ceftriaxone. *Acinetobacter* spp. was 100% resistant to Tetracycline in hospital-acquired UTI. Penicillin and Ampicillin were 100% resisted by *Enterococcus* spp. in community-acquired urinary tract infections. (83.3%) and (76.9%) of *E. coli* isolates were resistant to Ampicillin in hospital and community-acquired urinary tract infections, respectively. 83.3% vs. 37.5, 83.3 vs. 37.5, and 83.3% vs. 50% of CONs isolates in hospital and community-acquired urinary tract infection resist Penicillin, cotrimoxazole, and Tetracycline, respectively. 80% vs. 50% and 80% vs. 100% Augmentin and Tetracycline were resisted by *Klebsiella* spp. in hospital and community-acquired urinary tract infections, respectively (Table 5 and 6).

*E. coli*, *Proetus* spp., and *Klebsiella* spp. were susceptible to 100% for Meropenem in both community and hospital-acquired urinary tract infections, and *Citrobacter* spp., *Pseudomonas* spp., and *Acinetobacter* spp. were susceptible to 100% for Meropenem in hospital-acquired urinary tract infection. Even though the sample size is too small, *Proteus* spp., *Klebsiella* spp., *Citrobacter* spp., and *Enterococcus* spp. (%100) were susceptible to Nitrofurantoin. Meropenem was the most sensitive drug 100% for both types of urinary tract infection (Table 5 and 6).

Multidrug resistance (MDR) was detected in 18 (69.2%) and 11(61.1%) of *E. coli* isolates in both community and hospital-acquired urinary tract infections, respectively. The resistance patterns of 103 bacterial uropathogens isolated were tested against 15 antimicrobial agents. The predominant profile of *E. coli* MDR was observed in AMP, AMOX-CLAV, TET, and SXT with 4 (3.85%) among hospital-acquired and AMP, AUG, TET, CPR, SXT, and CTR with 2 (5.56%) among community-acquired UTI. Among the total numbers, *Proetus* spp. isolates were 100% MDR, three of them in hospital-acquired and the rest community-acquired. The scenario was also observed in *Klebsiella* spp. Moreover, *S. aureus* MDR was a higher proportion in hospital-acquired cases than community-acquired cases. In this study, antibiotic profile, except Penicillin, one *S. aureus* isolate resists all antimicrobials in hospital-acquired urinary tract infections (Table 7).

## 4. Discussion

Urinary tract infection is the most common infectious disease in both community and hospital-acquired settings. In this study, the overall prevalence of urinary tract infection was 23.7% (95%CI: 19.3–27.5), which lies between the low prevalence of (8.7%) in Iran [27], and the high prevalence of 90.1% Ethiopia-Shashemenie [28], in the different areas of the world. The results of this study also showed that the etiologic agents of UTIs mainly belonged more Gram-negative bacilli 61/103 (59.22%) than Gram-positive cocci 42/103 (40.78%). It is a known fact that Gram-negative isolates are the most prominent uropathogens compared to Gram-positive isolates, and the common source of pathogens causing UTI is intestinal flora which contains many Gram-negative organisms. Hence, the infection may be due to fecal contamination arising from poor hygiene [29].

The present study showed that the prevalence of CAUTI and HAUTI was 50 (19.7%) (95%CI:16.0–24.7) and 50 (30.7%) (95%CI:23.3–38), respectively. This prevalence was comparable with Nigeria, (36%) HAUTI [5] and Israel, 24.2% CAUTI [30]. But, lower than a study done in Northwestern India (44.27% vss 39.8%) [31], Saudi Arabia (55.3% vs. 44.7% [32], Bangladesh (45% vs. 50%) [33], Kuwait (59% vs. 41%) [34], Congo (35% vs. 65%) [21], and Yemen(51.2 vs. 48.8) [34] CAUTI and HAUTI, respectively, and higher than studies done in Nigeria (14.9% vs. 11.1%) [35], Southwestern Nigeria CAUTI (11%) [36], and Bahir Dar (9.4%) HAUTI (37). The reality might justify the variation that differences exist in the sample size of study participants, time of study period, study setting (community-based or hospital-based), characteristics of the population studied (age, residence, immunological status, urological disorders, socioeconomic status, educational level, hygiene practice), and laboratory methods.

According to our study, *E. coli* was the predominant uropathogen and no significant change has occurred in terms of pattern or position, of 26/50 (52%) and 18/53 (33.96%) in community and hospital-acquired UTI, respectively. This report is comparable with Nigeria [5], Saudi Arabia(38), and Kuwait [33]. It might be due to several virulent factors specific for colonization and invasion of the urinary epithelium, such as P-fimbria and S-fimbria adhesion. However, the frequency of isolation of *E. coli* in urine samples varies in diﬀerent study areas. It may be due to the high variation of different species of bacteria in the study and differ in the laboratory method of isolation. This makes it difficult to compare.

The frequency of CAUTI caused by *E. coli* is higher than that of HAUTI in this study. This is because most of the bacterial organisms causing UTI originate from the fecal flora and are dominated by various virulence factors that facilitate the ascent of bacteria from the perianal area to the urethra into the bladder and less frequently allow the organisms to reach the kidneys to induce symptomatic inflammation [38].

The second most frequent bacteria isolated were *S. aureus* at isolation rates of (24% vs. 24.5%) in CAUT and HAUTI, respectively. This result was a similar pattern with the study in Arbaminch, Ethiopia [29], and Southwestern Nigeria [39] in CAUTI and HAUTI, respectively. This study was at variance with other studies that reported a higher prevalence of other Gram-negative enteric bacilli Congo [21] *K. pneumoniae*, Saudi Arabia [32] *Enterobacter* spp., and Bangladesh [33] *Pseudomonas* spp. compared to as *S. aureus*.

In our study, *coagulase-negative staphylococcus* was the third position 8/50(16%) and 6/53 (11.3%) for CAUTI and HAUTI, respectively. However, our result was not correlated with other reports in Saudi Arabia [32], Bangladesh [33], and Abuja Nigeria [5] *Pseudomonas* spp. and is a retrospective study in Dessie regional lab, the second and third isolates were *Klebsiella* spp. and *Proteus* spp., respectively [40]. The possible reason for this variance was most Gram-positive bacteria survived commensally and it has been shifting with the environmental conditions such as temperature, humidity and resistance patterns. On the other hand, an increase in *Staphylococcal* UTI in the hospital setup may increase the use of instrumentation such as a catheter [41].

In our study, the prevalence of *Klebsiella* spp. was higher in hospital-acquired setting 5/53 (9.4%) than in community-acquired setting 2/50 (4%). In this study, it can be justified by its ability for adaptation to the hospital environment, and it can survive longer than other bacteria, enabling cross-infection within hospitals [42]. This report is correlated with the study in Bangladesh [33]. *Citrobacter* spp.*, Pseudomonas* spp., and *Acinetobacter* spp. were isolated from hospitalized patients only. Similar results were found in Yemen [34]. *Pseudomonas* spp. is enabled to survive and thrive well in soaps and disinfectants used for urethral catheterization [35]. Antimicrobial resistance has been recognized as an emerging worldwide problem in both ideveloped and developing countries. In hospital-acquired urinary tract infections, the resistance rate of Gram-negative isolates was 86.6% and 75% for Ampicillin and Amox-clav, respectively. In Gram-positive isolates, 76% and 68.4% were resistant to Tetracycline and SXT, respectively, except *Enterococcus* spp. This result was comparable with Yemen [37].

In community-acquired urinary tract infections, resistance for Ampicillin and Tetracycline was 79.3% and 68.9% to Gram-negative isolates and 61.9% and 36% for Tetracycline and SXT to Gram-positive isolates, respectively. This report is in agreement with Jordan (73%) [43] and Yemen (tetracycline (68.7%)) [34].The high proportion of resistance found in ampicillin, tetracycline, amox-clav, and SXT in both settings could be explained by the long period for which these drugs have been available and in use for UTI and in our study area, people having easy access to antibiotics in drug shops and therefore greater intake that contributes to increased the proportion of resistance. However, meropenem and nitrofurantoin were the most active drugs for both types of UTI because they are not easily accessible. This report was similar to Northwestern India for Nitrofurantoin [31].

In this study, the overall prevalence of multidrug resistance was 55.3% (57/103) (95%CI:10.0–16.8), in which 72.2% (21/29) of Gram-negative and 20% (4/20) of Gram-positive isolates in CAUTI and 75% (21/28) of Gram-negative and 57% (11/19) of Gram-positive isolates in HAUTI were observed; this finding showed that Gram-negative isolates were almost equally distributed. In both settings, this may be due to multidrug resistance bacteria circulating from hospital setting to community setting and vice versa. On the other hand, almost twofold Gram-positive MDR isolates were seen in hospital setup; most probably due to instrumentation or unsafe healthcare practice. This report was lower than the study in Gondar [44]. The possible reason for this result was geographical variation. Different demographic characteristics in various studies have been described to be associated with an increase in community and hospital-acquired UTI. In our study on CAUTI, previous use of antibiotics was significantly associated (*P*=0.024) with the prevalence of CAUTI. Of 39.4% of the study subjects who had previously used antibiotics for UTI or other than UTI, 36.3% were culture-positive. This finding was consistent with other studies, India [45] in Gondar, Ethiopia [46]. The possible reason for this finding is that the common source of pathogens causing UTI is intestinal flora exposed to too many antibiotic classes for UTI and other than UTI bacterial diseases, and hence damaging intestinal flora, then allowing uropathogens to colonize and subsequently infect the urinary tract [47]. In the previous retrospective study, Dessie regional lab, being female, and age were risk factors of developing CAUTI as compared to males. But in this study, none of them were associated with these infections. The differences observed in this study might be because of characteristics of the population studied (immunological status, urological disorder), and most isolates from the community that is tested in the regional lab may be predominantly from referral patients for whose previous antimicrobial treatment failed.

In HAUTI, patient setting, sex, age, diabetic mellitus, catheterization, and previous use of antibiotics have a statistically significant relationship with significant bacteriuria. Similar studies were found in Gondar-Ethiopia [46], Harar-Ethiopia [36], and Uganda [48]. The high prevalence of bacteriuria among inpatients 53/103(51.5%) as compared to the outpatients, 50/103 (48.5%) was that increased risk of infection due to indwelling catheter that contributed to 64.4% of the inpatients UTIs. (*X*2 = 6.537, *P*=0.011). This study is comparable with the study conducted in Uganda (49) and India [45] because infections could have been acquired through unsafe healthcare practices such as catheterization.

In the present study, the difference in the incidence of HAUTI among the males was 16.7% (9/54), and females 37.6% (41/109) was statically significant (*P*=0.008). This indicates that females have stronger predictions for HAUTI compared to males. The possible reason for this finding is females are more catheterized than males due to obstetric and gynecological causes (urinary tract abnormalities or obstruction) and shorter length of female urethra, its proximity to the anal canal, and absence of prostatic secretions. This report is similar to the study in Bangladesh [33].

In this study, the age range of 15–29 years, the isolation rate of CAUTI and HAUTI were 91% in females and 8.7% in males and 0% male, and 100% in females, respectively. This high frequency is due to the sexually active stage in females or probably pregnant women. On the other hand, old age (>60 years) 50% female and 50% male and 29.2% in male and 70.8% in female in CAUTI and HAUTI were found, respectively. The high isolation rate of UTI among the old age group could be due to Genito-urinary atrophy and vaginal prolapse after menopause which increasing vaginal pH and decreasing vaginal Lactobacillus that allows Gram-negative bacteria to grow and act asuropathogens [33]. Moreover, it is the possible reason for males' prostatic gland enlargement and decrease of bacteriostatic prostatic secretions might account for such infections [33]. This finding is comparable with Bangladesh [33], Nigeria [49], and Shashemenie in Ethiopia [28].

In hospital-acquired urinary tract infections, previous use of antibiotics was significantly associated (*P*=0.003) with the prevalence of hospital-acquired urinary tract infections. Of the total of 31.9% of previous antibiotic users for UTI or other than UTI, 52% of them were culture-positive. Our studies reflected that the prior and continuous use of antibiotics correlates with the UTI because the widespread use of antibiotics may cause multiple drug resistance microorganisms, this finding correlated with the study conducted in India [50] and Bangladesh [33].

In this study, urinary catheterization was the leading one among the causes of UTI due to instrumentation. From this study, 31.2% of UTI symptomatic study subjects used catheters in the hospital settings, 64.4% of those study participants were culture-positive. This reflects the greater proportion of HAUTI was significantly associated (*P*=0.014) with catheterization. This finding is comparable with the study in Bangladesh [33], India [45], and Bahir Dar-Ethiopia [48].

The statistically significant association between HAUTI and diabetes (*P*=0.002) could be due to altered immunity in diabetic patients, which includes depressed polymorphonuclear leukocyte functions, altered leukocyte adherence, chemotaxis, phagocytosis, the impaired bactericidal activity of the antioxidant system [51], and neuropathic complications, such as impaired bladder emptying. Moreover, a higher glucose concentration in the urine may create a culture medium for pathogenic microorganisms in diabetic patients that may result in this UTI. Similar reports are shown in Harar-Ethiopia [36], Nepal [52] India [45], and Uganda [48].

## 5. Conclusion and Recommendations

Findings of this study revealed that the overall prevalence rate of UTI was slightly high, and the hospital-acquired UTI group of patients has a higher rate of infection than community-acquired infection. *E. coli* is still the leading cause of community and hospital-acquired UTI, along with its increasing resistance pattern to different antibiotics, and is going to be an alarming health hazard. This study has shown that the alarming level of resistance (Ampicillin) achieved by bacteria is involved in causing UTI. *E. coli* and various isolates were more sensitive to meropenem and nitrofurantoin compared to other antibiotics tested.

The healthcare policy should be discouraging inappropriate use of antibiotics and prevent further development of resistant strains among bacteria. A continual audit of antimicrobial susceptibility patterns among the community and hospital-acquired UTI as a cause of morbidity should be performed and the findings should be reviewed periodically. Awareness should be created among the community members to prevent risk factors associated with the infection. Nitrofurantoin should be the first choice for empirical treatment of UTI in this study area. Further research should be focused on the effectiveness of risk factor reducing strategies and the changes to economic costs and healthcare benefits.

## 6. Limitation of the Study

This study did not consider anaerobic bacteria and few bacterial isolates were not identified at the species level that causes UTI due to lack of facility. Our study limitation was using only the antibiotics disks diffusion method to perform antimicrobial susceptibility instead of the microdilution method.

## Figures and Tables

**Table 1:** Sociodemographic characteristics of the study participant among the community and hospital-acquired UTI at Dessie Referral Hospital.

Characteristics (*n* = 422)		Negative, *N* (%)	Positive, *N* (%)	Total, *N* (%)
Sex	Male	122 (86.5)	19 (13.5)	141 (33.4)
	Female	200 (76.7)	81 (23.3)	281 (66.6)

Age	0–14	55 (83.3)	11 (16.6)	66 (15.6)
	15–29	77 (70)	33 (30)	110 (26)
	30–44	96 (84.2)	18 (15.7)	114 (27)
	45–59	60 (83.3)	12 (16.7)	72 (17)
	>60	34 (56.6)	26 (43.3)	60 (14.2)

Education	Illiterate	89 (71.7)	35 (28.2)	124 (29.4)
	Read and write only	106 (77.4)	31 (22.6)	137 (32.4)
	Up to grades 8 complete	86 (81)	20 (19)	106 (25)
	Up to grade 12 complete	17 (70)	7 (29)	24 (5.6)
	University/college and above	24 (77.4)	7 (22.6)	31 (7.3)

Residence	Urban	185 (77.7)	53 (22.3)	238 (56.4)
	Rural	137 (72.8)	51 (27.2)	188 (43.6)

Monthly income	Lower (<500 EBR)	150 (70)	64 (30)	214 (50.7)
	Medium (5001–1000)	106 (83.4)	21 (16.6)	127 (30)
	Higher (>1001 EBE)	66 (81)	15 (8.5)	81 (19.3)

Pregnancy status (*n* = 199)	Yes	10 (50)	10 (50)	20 (10)
	No	136 (78.8)	43 (21.2)	179 (90)

Diabetics (CDs)	Yes	29 (40.2)	43 (59.7)	72 (17)
	No	293 (83.7)	57 (16.3)	350 (83)

History of urinary tract obstruction	Yes	17 (42.5)	23 (57.5)	40 (9.5)
	No	305 (80.9)	77 (19.1)	382 (90.5)

Previous exposure to antibiotics	Yes	90 (58.4)	64 (41.5)	154 (36.4)
	No	232 (86.5)	36 (13.4)	268 (63.6)

Recurrence urinary tract infection	Yes	107 (64)	60 (36)	167 (40)
	No	215 (89.1)	40 (10.9)	255 (60)

History of renal calculi	Yes	38 (77.5)	11 (22.4)	49 (11.6)
	No	303 (81.4)	70 (18.6)	373 (88.4)

Contraceptive method (*n* = 253)	Yes	16 (84.2)	3 (15.8)	19 (7.5)
	No	178 (78.3)	56 (21.7)	234 (92.5)

Waiting time in hospital (*n* = 163)	48–72 hours	45 (80.4)	11 (19.6)	56 (34.3)
	>72 hours	68 (63.6)	39 (36.4)	107 (65.7)

History of catheterization (*n* = 163)	Yes	21 (41.2)	30 (58.8)	51 (31.2)
	No	92 (82.1)	20 (17.9)	112 (68.8)

Duration of catheterization (*n* = 53)	<One week	8 (53.3)	7 (46.7)	15 (28.3)
	>One week	13 (34.2)	25 (65.8)	38 (71.7)

**Table 2:** Distribution of culture among community versus hospital-acquired UTI groups of patients at Dessie Referral Hospital, Dessie.

UTI types	Urine cultured	Cultured positive cases	Percentage	*X* ^2^	*P*
CAUTI	259	50	19.3	6.537	0.011
HAUTI	163	50	30.7		
Total	422	100	23.7		

*Note:* by chi-squire, *X*^2^ = 6.537, df = 1, *P*=0.011; HAUTI: hospital-acquired UTI; CAUTI: community-acquired UTI.

**Table 3:** Distribution of bacterial isolated among CAUTI and HAUTI at Dessie Referral Hospital Dessie.

Isolated organism	Type of UTI				
	CAUTI (*n* = 50)		HAUTI (*n* = 53)		Total
	Male	Female	Male	Female	
*Escherichia coli*	5 (19.2)	21 (80.8)	4 (22.2)	14 ( (77.8)	44 (42.72)
*S. aureus*	3 (25)	9 (75)	3 (23)	10 (77)	25 ( (24.27)
CONS	1 (12.5)	7 (87.5)	1 (16.7)	5 (83.3)	14 (13.6)
*Klebsiella* spp.	1 (50)	1 (50)	1 (20)	4 (80)	7 (6.78)
*Proteus* spp.	0 (0)	1 (100)	0 (0)	3 (100)	4 (3.88)
*Enterococcus* spp.	0 (0)	1 (100)	0 (0)	2 (100)	3 (2.91)
*Pseudomonas* spp.	0 (0)	0 (0)	0 (0)	3 (100)	3 (2.91)
*Citrobacter* spp.	0 (0)	0 (0)	0 (0)	2 (100)	2 (1.94)
*Acinetobacter* spp.	0 (0)	0 (0)	0 (0)	1 (100)	1 (0.99)
Total	10 (20)	40 (80)	9 (17)	44 ( (83)	103 (100)

*Note: CONs* *=* coagulase-negative *Staphylococcus,* HAUTI *=* hospital-acquired UTI, CAUTI *=* community-acquired UTI.

**Table 4:** Bivariate and multivariate analyses of risk factors associated with community and hospital-acquired UTI among Dessie Referral Hospital.

Variable	Hospital-acquired				Community-acquired			
	Corollary (95% CI)	*P* value	AOR (95%CI)	*P* value	Corollary (95% CI)	*P* value	AOR (95%CI)	*P* value
*Sex*								
Male	1				1			
Female	3.01 (1.336–6.803)	0.008	8.93 (1.79–44.5)	0.008	2.3 (1.10–4.94)	0.026	0.4 (0.072–2.06)	0.266

*Age*								
0–14	0.17 (0.05–0.581)	0.005	0.33 (0.045–2.5)	0.280	0.85 (0.21–3.3)	0.811		
15–29	0.17 (0.063–0.463)	≤0.001	0.13 (0.02–0.79)	0.027	2.8 (0.86–9.13)	0.085	3 (0.476–19.24)	0.240
30–44	0.06 (0.018–0.218)	≤0.001	0.06 (0.06–0.6)	0.008	1.3 (0.37–4.25)	0.707		
45–59	0.27 (0.094–0.791)	0.017	0.26 (0.04–1.85)	0.180	0.4 (0.081–1.9)	0.247		
>60	1				1			

*Education*								
Illiterate	1.29 (0.291–5.766)	0.733			1.5 (0.44–4.82)	0.553		
Read and write only	1.23 (0.287–5.233)	0.784			0.644 (0.18–2.3)	0.499		
1–8 grade complete	0.60 (0.129–2.794)	0.515			0.92 (0.26–3.26)	0.893		
Up to grade 12 complete	1.17 (0.133–10.22)	0.889			1.63 (0.3657.36)	0.521		
University/college	1				1			

*Residence*								
Urban	1				1			
Rural	1.71 (0.874–3.374)	0.117	2.93 (0.969–8.88)	0.057	1.26 (0.86–2.33)	0.459		

*Monthly income*								
Lower (<500EBR)	1.47 (0.584–3.719)	0.412			1.484 (0.65–3.4)	0.353		
Medium (5001–1000EBR)	0.61 (0.203–1.821)	0.374			1.1 (0.449–2.75)	0.820		
Higher (>1001EBE)	1				1			

*Pregnancy status*								
Yes	2.39 (0.54–10.612)	0.252			3.72 (1.1–12.53)	0.034	3.340 (0.78–14.10)	0.102
No	1				1			

*Diabetics (CDs)*								
Yes	9.02 (4.130–19.71)	≤0.001	6.70 (1.994–22.53)	0.002	5.243 (2.3–11.9)	≤0.001	1.751 (0.47–6.49)	0.402
No	1				1			

*History of urinary tract obstruction*								
Yes	6.10 (2.082–13.85)	≤0.001	2.69 (0.712–10.18)	0.144	1.650 (.312–8.8)	0.553		
No	1				1			

*Previous exposure to antibiotics*								
Yes	4.13 (2.028–8.420)	≤0.001	5.69 (1.84–17.59)	0.003	5.8 (2.944–11.5)	≤0.001	4.427 (1.21–16.15)	0.024
No	1				1			

*Recurrence urinary tract infection*								
Yes	4.65 (2.229–9.701)	≤0.001	1.62 (0.499–5.23)	0.423	3.4 (1.75–6.604)	≤0.001	1.023 (0.31–3.36)	0.970
No	1				1			

*History of renal calculi*								
Yes	6.17 (1.154–32.96)	0.033	9.47 (0.58–153.3)	0.114	1.65 (0.61–0.23)	0.331		
No	1				1			

*Use of Contraceptive method*								
Yes	0.000 (0.000---)	0.999			0.9 (0.24–3.402)	0.880		
No	1				1			

*Waiting time in hospital*								
48–72 hours	1							
>72 hours	2.35 (1.089–5.056)	0.029	2.35 (0.77–7.18)					

*History of catheterization*								
Yes	6.92 (3.284–14.57)	≤0.001	3.9 (1.323–11.5)					
No	1							

*Duration of catheterization*								
<One week	1							
>One week	2.20 (0.652–7.413)	0.204						

*Note.* COR: crude odd ratio; AOR: adjusted odd ratio; CI: confidence interval; EBR: Ethiopian birr; 1: reference.

**Table 5:** Antimicrobial susceptibility pattern of hospital-acquired urinary tract infection at Dessie Referral Hospital, Dessie.

Isolates (*N* = 53)	Antimicrobial agent, *N* (%)											
Gram-positive		CIP	TE	F	SXT	CN	*P*	AMP	VA			
*S. aureus* (*n* *=* 13)	S	9 (69.2)	2 (15.4)	9 (60.2)	5 (38.5)	10 (77)	4 (33.3)	ND	ND			
	I	0 (0)	2 (15.4)	0 (0)	0 (0)	1 (7.7)	1 (8.3)					
	R	4 (30.8)	9 (69.2)	4 (30.8)	8 (61.5)	2 (15.4)	7 (58.3)					

*CONS* (*n* *=* 6)	S	3 (50)	1 (16.7)	4 (66.7)	1 (16.7)	4 (66.7)	1 (16.7)	ND	ND			
	I	1 (16.7)	0 (0)	0 (0)	0 (0)	0 (0)	0 (0)					
	R	2 (33.3)	5 (83.3)	2 (33.3)	5 (83.3)	2 (23.3)	5 (83.3)					

*Enterococcus* spp. (*n* *=* 2)	S	1 (50)	0 (0)	2 (100)	ND	ND	1 (50)	1 (50)	2 (100)			
	I	0 (0)	0 (0)	0 (0)			0 (0)	0 (0)	0 (0)			
	R	1 (50)	2 (100)	0 (0)			1 (50)	1 (50)	0 (0)			

Gram-negative		CIP	T	F	SXT	CN	AMP	MEM	CRO	NA	AMC	CAZ
*E. coli* (*n* = 18)	S	12 (66.7)	5 (29)	16 (88.9)	8 (44.4)	12 (66.7)	1 (5.6)	18 (100)	15 (83.3)	15 (83.3)	5 (27.8)	12 (66.7)
	I	0 (0)	2 (11)	1 (5.6)	0 (0)	1 (5.6)	2 (11.1)	0 (0)	0 (0)	0 (0)	0 (0)	0 (0)
	R	6 (33.3)	11 (61)	1 (5.6)	10 (55.6)	5 (27.7)	15 (83.3)	0 (0)	3 (16.7)	3 (16.7)	13 (72.2)	6 (33.3)

*Proteus* spp. (*n* = 03)	S	3 (100)	0 (0)	3 (100)	0 (0)	1 (33.3)	0 (0)	3 (100)	2 (66.7)	3 (100)	1 (33.3)	2 (66.7)
	I	0 (0)	0 (0)	0 (0)	0 (0)	0 (0)	0 (0)	0 (0)	0 (0)	0 (0)	0 (0)	0 (0)
	R	0 (0)	3 (100)	0 (0)	3 (100)	2 (66.7)	3 (100)	0 (0)	1 (33.3)	0 (0)	2 (66.7)	1 (33.3)

*Klebseilla* spp. (*n* = 5)	S	3 (60)	0 (0)	5 (100)	2 (40)	3 (60)	0 (0)	5 (100)	2 (40)	4 (80)	0 (0)	4 (80)
	I	1 (20)	1 (20)	0 (0)	0 (0)	0 (0)	0 (0)	0 (0)	0 (0)	1 (20)	1 (20)	0 (0)
	R	1 (20)	4 (80)	0 (0)	3 (60)	2 (30)	5 (100)	0 (0)	3 (60)	0 (0)	4 (80)	1 (20)

*Citrobacter* spp. (*n* = 2)	S	1 (50)	0 (0)	2 (100)	0 (0)	1 (50)	0 (0)	2 (100)	0 (0)	1 (50)	0 (0)	1 (50)
	I	1 (50)	0 (0)	0 (0)	0 (0)	1 (50)	0 (0)	0 (0)	0 (0)	1 (50)	0 (0)	0 (0)
	R	0 (0)	2 (100)	0 (0)	2 (100)	0 (0)	2 (100)	0 (0)	2 (100)	0 (0)	2 (100)	1 (50)

*Pseudomonas* spp. (*n* = 3	S	0 (0)	ND	ND	ND	2 (66.7)	ND	3 (100)	ND	ND	ND	ND
	I	1 (33.3)				0 (0)		0 (0)				
	R	2 (66.7)				1 (33.3)		0 (0)				

*Acetobacter* spp. (*n* = 1)	S	1 (100)	0 (0)	ND	0 (0)	0 (0)	ND	1 (100)	ND	ND	ND	ND
	I	0 (0)	1 (100)		0 (0)	1 (100)		0 (0)				
	R	0 (0)	0 (0)		1 (100)	0 (0)		0 (0)				

Total	S	33 (37.4)	8 (16)	41 (83.6)	15 (33)	33 (64.7)	7 (15)	33 (97)	21 (70)	23 (82)	6 (21)	19 (68)
	I	5 (9.4)	6 (4)	1 (2)	0 (0)	4 (7.8)	3 (6)	0 (0)	0 (0)	2 (7)	1 (4)	0 (0)
	R	15 (28)	36 (80)	7 (14.4)	32 (67)	14 (27.4)	38 (79)	1 (3)	9 (30)	3 (11)	21 (75)	9 (32)

Note: AMP, ampicillin; CIP, ciprofloxacin; CRO, ceftriaxone; AMC, amoxicillin-clavulanate; CN, gentamicin; F, nitrofurantoin; SXT, cotrimoxazole; NA, nalidixic acid; CAZ, ceftazidime; MEM, meropenem; VA, vancomycin; TE, tetracycline; ND, not done.

**Table 6:** Antimicrobial susceptibility pattern of community-acquired urinary tract infection at Dessie Referral Hospital, Dessie.

Isolates(*N* = 50)	Antimicrobial agent, *N* (%)											
Gram-positive		CIP	TE	F	SXT	CN	*P*	AMP	VA			
*S. aureus (n* *=* 12)	S	8 (66.7)	1 (8.3)	12 (100)	5 (41.7)	9 (75)	7 (58.3)	ND	ND			
	I	0 (0)	3 (25)	0 (0)	1 (8.3)	0 (0)	1 (8.3)					
	R	4 (33.3)	8 (66.7)	0 (0)	6 (8.3)	3 (25)	4 (33.3)					

*CONS (n* *=* 8)	S	6 (75)	3 (37.5)	7 (87)	5 (62.5)	7 (87.5)	5 (62.5)	ND	ND			
	I	1 (12.5)	1 (12.5)	1 (12.5)	0 (0)	0 (0)	0 (0)					
	R	1 (12)	4 (50)	0 (0)	3 (37.5)	1 (12.5)	3 (37.5)					

*Enterococcus* spp. *(n* *=* 1)	S	1 (100)	0 (0)	1 (100)	ND	ND	0 (0)	0 (0)	0 (0)			
	I	0 (0)	0 (0)	0 (0)			0 (0)	0 (0)	1 (100)			
	R	0 (0)	1 (100)	0 (0)			1 (100)	1 (100)	0 (0)			

Gram-negative		CIP	T	F	SXT	CN	AMP	MEM	CRO	NA	AMC	CAZ
*E. coli* (*n* = 26)	S	24 (92.3)	9 (34.5)	25 (96.2)	12 (46.2)	21 (80.8)	5 (19.2)	26 (100)	21 (80.8)	18 (69.2)	11 (42.3)	22 (84.6)
	I	1 (3.8)	0 (0)	1 (3.8)	0 (0)	1 (3.8)	1 (3.8)	0 (0)	2 (7.7)	0 (0)	1 (3.8)	2 (3.8)
	R	1 (3.8)	17 (65.4)	0 (0)	14 (53.8)	4 (15.4)	20 (76.9)	0 (0)	3 (11.5)	8 (30.8)	14 (53.8)	3 (11.5)

*Proteus* spp. (*n* = 1)	S	1 (100)	0 (0)	1 (100)	0 (0)	0 (0)	0 (0)	1 (100)	1 (100)	1 (100)	0 (0)	1 (100)
	I	0 (0)	0 (0)	0 (0)	0 (0)	1 (100)	0 (0)	0 (0)	0 (0)	0 (0)	0 (0)	0 (0)
	R	0 (0)	1 (100)	0 (0)	1 (100)	0 (0)	1 (100)	0 (0)	0 (0)	0 (0)	1 (100)	0 (0)

*Klebsiella* spp. (*n* = 2)	S	1 (50)	0 (0)	2 (100)	1 (50)	1 (50)	0 (0)	1 (100)	1 (50)	1 (50)	1 (50)	1 (50)
	I	0 (0)	0 (0)	0 (0)	0 (0)	0 (0)	0 (0)	0 (0)	0 (0)	0 (0)	0 (0)	0 (0)
	R	1 (50)	2 (100)	0 (0)	1 (50)	1 (50)	2 (100)	0 (0)	1 (50)	1 (50)	1 (50)	1 (50)

Total	S	41 (82)	13 (26)	48 (96)	23 (48)	38 (76)	18 (36)	28 (93)	23 (77)	20 (69)	12 (41.4)	24 (80)
	I	2 (4)	4 (8)	2 (4)	1 (2)	2 (4)	2 (4)	0 (0)	3 (10)	0 (0)	1 (3.4)	2 (7)
	R	7 (14)	33 (66)	0 (0)	25 (50)	9 (20)	30 (60)	1 (7)	4 (13)	9 (31)	16 (55)	4 (13)

Note: AMP, ampicillin; CIP, ciprofloxacin; CRO, ceftriaxone; AMC, amoxicillin-clavulanate; CN, gentamicin; F, Nitrofurantoin; SXT, cotrimoxazole; NA, nalidixic acid; CAZ, ceftazidime; MEM, meropenem; VA, vancomycin; TE, tetracycline; ND, not done.

**Table 7:** Multidrug resistance pattern of community and hospital-acquired bacterial isolates among Dessie Referral Hospital UTI suspected patients at Dessie, March-April, 2019.

Isolates	Community-acquired isolates		Hospital-acquired isolates	
	Antimicrobial agents	N (%)	Antimicrobial agents	*N* (%)
*E. coli* [18]*-*IPD	AMC, SXT, CXM	1 (3.85)	AMP, AMOX-CLA, GEN	1 (5.56)
*E. coli* [26]-OPD	AMP, AMOX-CLAV, SXT, CTR	1 (3.85)	AMP, AMOX-CLAV, TET, SXT, CXM	1 (5.56
	NAL, SXT, CTR	1 (3.85)	AMOX-CLAV, SXT, CXM	1 (5.56)
	AMP, AMOX-CLAV, TET	2 (3.85)	AMP, AMOX-CLAV, TET CPRSXT, CXM	1 (5.56)
	AMP, AMOX-CLAV, TET, SXT	4 (3.85)	AMP AMOX-CLAV CPR GEN, NIT	1 (5.56)
	TET, SXT, GEN	1 (3.85)	AMP, TET, CPR	1 (5.56)
	AMP, AMOX-CLAV NAL, TET, GEN	1 (3.85)	AMP, NAL, TET	1 (5.56)
	AMP, NAL, TET	1 (3.85)	AMP, TET, CPR, SXT, CTR	2 (5.56)
	AMP, AMOX-CLA, NAL TET, SXT	2 (3.85)	AMP, AMOX-CLAV, CPR	1 (5.56)
	AMP, AMOX-CLAV NAL, SXT	1 (3.85)	AMP AMOX-CLAV, SXT	1 (5.56)
	AMP, TET, SXT	1 (3.85)		
	AMP, TET, SXT, GEN	1 (3.85)		
	AMOX-CLAV, SXT	1 (3.85)		

Total		18 (69.2)		11 (61.1)

P*roetus* spp. [3]-IPD	AMP, AMOX-CLAV, TET, SXT	1 (100)	AMP, TET, SXT, GEN	1 (33.3)
*Proteus* spp. (1)-OPD			AMP, AMOX-CLAV, TET SXT	1 (33.33)
			AMP, AMOX-CLAV, TET, SXT, GEN, CTR	1 (33.33)

Total		1 (100)		3 (100)

*Klebsiella* spp. [5]-IPD	AMP, NAL, TET, SXT, GEN, CTR	1 (50)	AMP, AMOX-CLAV, TET	1 (20)
*Klebsiella* spp. [2]- OPD	AMP, AMOX-CLAV NAL, TET, CPR	1 (50)	AMP, AMOX-CLV SXT, GEN, CTR	1 (20)
			AMP, AMOX-CLAV, TET, CTR	1 (20)
			AMP, NAL, TET, CPR, SXT, CTR	1 (20)
			AMP, AMOX-CLAV, SXT	1 (20)

Total		2 (100)		5 (100)

*Citrobacter* spp [2]			AMOX-CLAV, AMP, NAL, SXT, GEN, CTR	1 (50)
			AMP, AMOX-CLAV, TET, SXT, CTR	1 (50)

Total				2 (100)

*S. aureus* [13]-IPD	TET, CPR, PEN, GEN	1 (8.3)	TET, SXT, PEN	3 (7.7)
*S. aureus* [12]-OPD	TET, CPR, SXT, PEN	1 (8.3)	TET, CPR, SXT, GEN, NIT	1 (7.7)
			TET, CPR, SXT, PEN	1 (7.7)
			CPR, SXT, PEN	1 (7.7)
			TET, PEN, NIT	1 (7.7)
				7 (53.9)

Total		2 (16.6)		

CONS [6]-IPD	TET, CPR, SXT	1 (12.5)	TET, PEN, GEN, CPR	1 (16.6)
CONS [8]-OPD	TET, PEN, GEN	1 (12.5)	TET, SXT, PEN	1 (16.6)
			TET SXT, GEN	1 (16.6)
			TET, CPR, SXT, PEN, GEN	1 (16.6)
Total		2 (25)		4 (66.7)

Note: IPD = inpatient, OPD = outpatient department, CONs = coagulase-negative *Staphylococcus*, AMP = ampicillin, TET = tetracycline, PEN = penicillin, GEN = gentamycin, CPR = ciprofloxacillin, SXT = sulphamethoxazole-trimethoprim, NIT = nitrofurantoin, CTR = ceftriaxone, CXM = cefixime.

## Data Availability

Data used to support the finding of this study are available from the corresponding author upon request.

## Abbreviations

ATCC: American Type Culture Collection
CAUTI: Community-acquired urinary tract infection
CFU: Colony forming unit
CLSI: Clinical and Laboratory Standard Institute
CONS: Coagulase-negative *Staphylococci*
HAUTI: Hospital-acquired urinary tract infection
HCWs: Healthcare workers
HPF: High power field
IPD: Inpatient department
OPD: Outpatient department
SOP: Standard operating procedure
SPSS: Statistical Package for Social Sciences
UTI: Urinary tract infection
WHO: World Health Organization.
